# ‘Not feeling heard’ in health care: A critical review of the detrimental effects of poor-quality listening

**DOI:** 10.1177/13591053251377544

**Published:** 2025-10-21

**Authors:** Gillian King

**Affiliations:** 1 Bloorview Research Institute, Holland Bloorview Kids Rehabilitation Hospital, Toronto, ON, Canada

**Keywords:** communication, engagement, inferences, motivation, negative experiences, negative outcomes, reflection, rehabilitation

## Abstract

Listening research focuses on the benefits of good-quality listening rather than the detrimental effects of poor-quality listening on the speaker. The aim of this critical review was to identify what is known about the effects of poor-quality listening in the fields of communication, the workplace, and health care, and to synthesize this knowledge to inform research and practice in health care. Based on the evidence, a multidimensional framework is proposed encompassing clients’ affective, cognitive, and behavioral reactions to poor-quality listening in health care, along with relational outcomes concerning the client and healthcare professional. This framework proposes three mechanisms underlying client reactions to perceived poor-quality listening—reflection, engagement, and motivation. When healthcare clients feel not listened to, this can have serious, wide-ranging, and cascading effects on their emotions, thoughts, and actions, leading to poor collaboration, poor-quality relationships with healthcare providers, and a lack of person-centered care.

## Introduction

Listening has been defined as a multidimensional construct comprising attention, understanding, and a relational perspective (Itzchakov and Kluger, 2017; Worthington and Bodie, 2018). From a relational point of view, the effects of listening are inherently related to the speaker’s perception of how a listener made them feel (Wolvin, 2010; Itzchakov and Kluger, 2017). Good-quality listening is, therefore, a perception arising from listener behaviors conveying attention, understanding, and positive intentions (Itzchakov et al., 2023). The intentions of the listener are essential to good listening (King, 2022), as it is hard to be a genuinely good listener without having a benevolent intention toward the speaker (Itzchakov et al., 2022). Nonetheless, the vast majority of people listen with the intention to reply rather than to understand, which can lead to negative reactions in speakers who feel ‘not heard’ (Kriz et al., 2021).

Despite the importance of listening and communication to human interactions, there has been little focus on listening from a theoretical point of view (Kluger and Itzchakov, 2022; Bodie, 2012). Existing theories of listening do not take sufficient account of the relational nature of listening (Kluger and Itzchakov, 2022) and the theoretical associations between listening and other phenomena, such as self-disclosure and trust, are often ignored (Bodie, 2012). As well, listening research has focused almost exclusively on the benefits of good listening on the speaker (Kluger and Itzchakov, 2022), rather than the effects of poor listening. This is important, as the negative effects of poor listening on speakers may not be benign (i.e., simply the absence of positive effects) but instead may be detrimental, including disempowerment, disengagement, damaged relationships, and poor collaboration. Poor listening is defined in this article as the speaker’s judgment that what they were trying to communicate was dismissed or disregarded and they themselves were not understood, validated, or valued (Hinz et al., 2022).

Although this article’s main interest concerns the implications of poor listening for health care recipients, initial literature searches indicated relatively little direct attention had been paid to poor listening in the healthcare context. Surprisingly little attention has been paid to the role of poor listening on patient care (Jagosh et al., 2011). Articles mention the phenomenon of poor-quality listening in passing, but typically focus on the benefits of listening. Consequently, a broader purview was taken in this article with respect to listening context, with particular interest in informing understanding of the effects of poor listening in health care. The following sections discuss how listening is conceptualized in this article, the nature of the health care context, and the negative experiences of healthcare clients who feel unheard.

### Conceptualization of listening

In this article, listening is conceptualized as a unitary construct comprising a bipolar continuum ranging from good to poor listening (positive and negative poles), with the neutral point (0) representing neither good nor poor listening. Thus, good listening reflects a judgment of engaged attention, understanding, and nonjudgmental intention, as would be displayed by good friends, intimate partners, and person-centred service providers or therapists. The neutral point (0) reflects ‘typical’ or ‘normal’ listening, as studied by Itzchakov et al. (2023), which is neither attentive/responsive nor considered dismissive, and does not lead to a perception of ‘not being heard’. Thus, the quality of listening is conceptualized along a graduated continuum.

Given this conceptualization of listening as a continuum, the outcomes of poor listening may not only be the opposite of those arising from good listening. Rather, poor listening potentially has qualitatively different effects. Borut et al. (2025) have argued that constructive and destructive listening (corresponding to good and poor listening, respectively) are separate unipolar constructs, as they have differential effects. However, according to Neff (2022), it is a fallacy to consider differential effects to be evidence of unipolarity. There is a long history of debate over the nature and effects of the opposite ends of a bipolar continuum (Neff, 2022), as well as evidence that a number of psychological constructs form bipolar continua, including job satisfaction/dissatisfaction (Kam and Meyer, 2015) and compassionate self-responding/uncompassionate self-responding (Neff, 2022). Thus, in this article, good and poor listening are considered to potentially have different outcomes, as stated by Neff (2022).

### Listening in the healthcare context

In health care, listening undergirds person-centered models of service, where the client’s needs and preferences are considered paramount, and care is characterized by good communication and listening, respect, trust, and client engagement (Byrne et al., 2020; King, 2022). When healthcare clients feel they are not properly listened to, negative experiences and outcomes can result, based on clients’ inferences, which refer to conclusions reached on the basis of evidence and reasoning. Based on their observations of healthcare professionals’ (HCPs’) behavior, clients make judgments about how well they have been listened to. For example, when physicians are perceived as being inauthentic in their listening, clients can infer that physicians do not care for their well-being (Jagosh et al., 2011).

To what extent is health care a unique communicative setting? First, although power differences between HCPs and clients may affect the conversations that take place, there also are power differences in employee-supervisor conversations in the workplace. Second, the outcomes of listening in health care include patient safety (e.g., medication errors and incorrect diagnoses), satisfaction, and health outcomes (Sharkiya, 2023), whereas the workplace literature focuses on job satisfaction, employee engagement, and job performance (Pery et al., 2020). Nonetheless, both literatures discuss the outcomes of miscommunication and engagement.

The aim of this critical review was, therefore, to identify what is known about the effects of poor-quality listening on speakers who do ‘not feel heard’ and to synthesize this knowledge to inform research and practice in health care.

## Methods

A critical review was conducted, following the analytical framework of Search, Appraisal, Synthesis, and Analysis (SALSA) (Grant and Booth, 2009). Diverse sources were searched to conduct a synthesis of information, generate new insights, and inform research and practice in health care (Grant and Booth, 2009). Critical reviews aim to provide a meaningful synthesis of literature on a complex topic, where a broad range of knowledge sources and methodologies exist (Greenhalgh et al., 2018), which is the case for listening research. Critical reviews identify the most relevant and significant publications on a topic (Samnani et al., 2017), examine their conceptual contributions to the literature (Grant and Booth, 2009), and emphasize interpretive synthesis rather than technical methods (Greenhalgh et al., 2018). In critical reviews, there is no formal requirement to present the methods in a systematic or explicit manner or to assess the quality of the retrieved publications (Grant and Booth, 2009). The critical review method was relevant to this paper, given the broad search for information on the effects of poor listening.

An ‘argument framework’ was also employed, which seeks to find whatever evidence is relevant (Greenhalgh et al., 2024) and makes use of inference, deductive validity, soundness (an argument that is valid), and confirmation (an argument based on evidence and reasoning) (Cartwright and Hardie, 2012). In the present case, the argument is that perceived poor-quality listening has a series of negative effects on the speaker who feels not listened to.

An expansive and iterative search was conducted to retrieve relevant literature, with no restriction on the years of publication. In January 2024, a preliminary Google Scholar search was conducted to capture peer-reviewed articles and book chapters on the negative effects or harms of poor clinical listening, and also on poor clinical outcomes associated with poor clinical listening skills. Colleagues were also asked to share articles on healthcare difficulties arising from poor-quality listening.

In February 2024, a second preliminary search was conducted in OVID Medline, OVID PsycINFO, Scopus, and CINAHL, with subject headings including listening, outcomes (health care), and communication skills. This search retrieved a systematic review and meta-analysis by Kluger and colleagues (2024) on the effects of perceived high-quality listening on work outcomes, which provided search terms (see below) to use in Scopus, EBSCO (CINAHL Plus with Full Text, Business Source Premier), and OVID.

With the advice of a health librarian, a third search was then conducted to identify peer-reviewed journal articles published in English that had poor w/3 listen* OR ineffective w/3 listen* in the article title, abstract, or keywords (*denotes the use of truncation; w/3 refers to proximity searching for two terms occurring within three words of each other). This search also involved looking at titles of articles citing Kluger et al.’s meta-analysis (2024). The search retrieved conceptual and empirical articles on listening, including articles on the effects, on speakers, of good and poor listening. Given the scant literature directly examining poor listening, article texts were hand searched for the mention of poor listening.

The set of over 100 retrieved articles was read in its entirety, noting cited articles and book chapters relevant to the topic, and retrieving these. At this point, further searches were conducted to identify articles providing more information on specific effects of poor listening and related concepts (e.g., disengagement, lack of psychological safety, cognitive inferences, and mechanisms and processes of change). Thus, threads in the literature were followed to delve into topics emerging from the articles that were read.

Tables were used extensively to organize information about pockets of literature, theories of listening, and affective, cognitive, and behavioral effects of good and poor listening. Using a process of interpretive synthesis, these tables were used to identify important aspects of the literature. A conceptual framework of client reactions to poor-quality listening in healthcare conversations was developed as a way of integrating the literature.

## Results

A Context-Mechanism-Outcome (CMO) framework, used in realist evaluation (Jagosh et al., 2015), is used to present the findings by Context (i.e., fields of literature), Outcomes (including outcomes of both good and poor listening, but with an emphasis on the latter), and Mechanisms (i.e., the unobservable yet real processes, sensitive to variations in context, that generate or influence outcomes) (Astbury and Leeuw, 2010).

### Fields of literature investigating listening

The review identified three fields of literature, namely listening in everyday interpersonal conversations (communication research), in conversations taking place in the workplace (workplace research), and in healthcare interactions.

#### Communication research

Communication research has focused on the behaviors or skills of the listener and the processes underlying listening (Wolvin, 2010). Various types of listening have been proposed, such as active, supportive, and active-empathic listening (Bodie, 2011; King, 2022). Theoretically, listening has been linked to positive communication (Bodie, 2012) and to the phenomenon of engagement (Wolvin, 2010). Research studies in this area are largely experimental, involving psychology students taking part in studies comparing listening under good listening conditions with ‘normal’ or ‘typical’ listening (Itzchakov et al., 2023; Pasupathi and Hoyt, 2010). ‘Normal’ or ‘regular’ listening was defined by Itzchakov et al. (2023) as moderate in quality (i.e., the listener maintained eye contact and provided nonverbal responses, as in a regular conversation).

Other studies have investigated the effects of poor listening. In an experiment involving undergraduate students, Itzchakov et al. (2023) manipulated the behaviors of poor listeners (e.g., leaning backwards, not providing verbal or nonverbal responses, and not asking questions) concluding that, in comparison to poor listeners, good listeners actively foster positive engagement. Lehmann et al. (2023) conducted several studies examining listening as an antecedent of humility, in which students were randomly assigned to good or poor listening conditions; poor listening was operationalized as distracted or argumentative listening. As well, Bavelas et al. (2000) investigated the effects of two types of listening responses (generic responses vs distracted listeners making fewer responses) on narrative story-telling. Narrators told their stories significantly less well when the listeners displayed signs of distraction. These studies by Itzchakov et al. (2023), Lehmann et al. (2023), and Bavelas et al. (2000) all involved unacquainted dyads.

In contrast to unacquainted dyads, Reis and Shaver (1988) considered the importance of the concept of responsiveness in communication in intimate relationships. According to their model of the intimacy process, a person feels a greater connection to their partner when they receive validating, understanding, and caring responses to self-disclosures, whereas non-responsive listeners are perceived as uncomprehending or uninterested. Consistent with disclosure-responsiveness theory, responsiveness to disclosures is associated with a decrease in deleterious effects and improved well-being (Reif-Stice et al., 2023).

#### Workplace research

The growing body of research on listening in the workplace has tended to adopt a relational perspective, considering the effects of good listening on the speaker. Theories in this organizational context include Episodic Listening Theory, which states that listening affects behavior in short episodes that may have lasting effects (Kluger and Itzchakov, 2022), and High Quality Connection Theory, which deals with short-term, dyadic, interactions that foster positive subjective experiences in the individuals involved (Stephens et al., 2011). Itzchakov and colleagues (Itzchakov et al., 2023; Kluger and Itzchakov, 2022) have investigated high-quality relationships or connections among speakers and listeners, considered to be the means by which positive workplace collaborations arise. Partner responsiveness is a key construct in this literature, with studies examining the effects of affective, cognitive, and behavioral reactions to partner responsiveness (Itzchakov et al., 2022). As mentioned previously, Kluger et al. (2024) conducted a meta-analysis of the effects of perceived high-quality listening on work outcomes, finding that perceived listening may enhance job performance through its effects on affect, cognition, and relationship quality. This literature has indicated that low-quality listening can lead to the disempowerment of the speaker (i.e., feeling their point of view is not heard, understood, or valued) (Hinz et al., 2022). As well, Kriz, Kluger, and Lyddy (2021) examined aspects of stories related to feeling heard (vs not) in employee-manager conversations, finding that employees became disengaged and disappointed when they felt unheard and managers took insufficient action.

#### Healthcare research

Research on listening in health care is fragmented but growing. There is some overlap between listening in healthcare and in the workplace, as the healthcare setting is one type of workplace. Many articles on listening in health care are reviews or thought pieces about the importance of good listening, stressing the idea of person-centered care and communication (King, 2022; McKenna et al., 2020). High-quality messages are “person-centered”, as they are tailored to the recipient’s viewpoint (Burleson and Rack, 2008). Listening in health care is considered essential to mutual engagement and other relational outcomes that mobilize clients towards pursuing goals (King, 2022). Articles have addressed topics such as health literacy, how patient-provider communication can affect the patient’s knowledge, motivation, engagement, empowerment, and health outcomes (Nouri and Rudd, 2015), and how patient-centered communication can improve patient satisfaction, adherence to treatment plans, and patient health (Charlton et al., 2008). According to Myers et al. (2020), client perceptions of not being listened to constitute a gap in the nursing literature.

The healthcare literature on listening includes reviews (King, 2022; Vermeir et al., 2015), qualitative studies (Jagosh et al., 2011), and intervention studies (King et al., 2017). These studies point not only to the importance of good listening, but also the harms of poor listening on person-centered care. For example, a qualitative study of the healthcare experiences of parents of children with disabilities indicated that parents were stressed and frustrated when they were not listened to by HCPs (King et al., 2025).

#### Summary

Articles on listening in the communication field are largely concerned with listener skills, listening processes, and the benefits of good-quality listening for the speaker, as ascertained in experimental studies involving psychology students who do not know one another (e.g., Wolvin, 2010; Gearhart and Bodie, 2011). In contrast, the workplace listening literature largely deals with conversations and relationships between employees and managers in real-life organizational settings, whereas the healthcare literature deals with client-HCP conversations in the context of service delivery. Recent articles on listening in the workplace and health care have taken the speaker’s perspective (e.g., Jagosh et al., 2011; Itzchakov et al., 2022), highlighting the importance of adopting a relational perspective on listening. Possibly reflecting the importance of a relational perspective to workplace collaborations and client-HCP interactions, more attention has been paid to poor-quality listening in these literatures.

The healthcare literature has pointed to a host of outcomes associated with good listening, including good quality care (Myers et al., 2020; McKenna et al., 2020), effective clinical practice (Jagosh et al., 2011), positive relationships between clients and healthcare professionals (HCPs) (Jagosh et al., 2011), and mutual client-HCP understanding, collaboration, and engagement (King, 2022). Last, studies of listening in the communication and workplace literature often use experimental designs, in contrast to the healthcare literature.

### Affective, cognitive, and behavioral aspects of listening

The literature review indicated the use of frameworks incorporating affect, behavior, and cognition (i.e., ABC frameworks) to conceptualize various aspects of good listening. Affect, cognition, and behavior are foundational elements of human experience; they provide important insights into how perceived poor-quality listening may affect the speaker.

ABC frameworks have been used to describe both the listener’s behavior and the speaker’s reactions to the quality of the listening. A multidimensional ABC view of listening processes has been proposed by communication researchers (Gearhart and Bodie, 2011; Bodie, 2012; Wolvin, 2010) and those investigating listening in the workplace (Itzchakov and Kluger, 2017). Listening has been conceptualized as an act signaling attention, understanding, and relating to the other person (Worthington and Bodie, 2018). These concepts are based on behavior, cognition, and affect, respectively. In addition, the speaker’s reactions to poor listening have been explicitly described in an ABC framework in the workplace literature (Itzchakov et al., 2022; Kluger et al., 2024) and, implicitly, in the listening of physicians in health care (Jagosh et al., 2011).

### Outcomes of poor listening for the speaker across fields of research

This section takes a comparative approach, integrating findings across the three fields of literature, and using an ABC framework to categorize the nature of the outcomes of poor listening. Table 1 presents a summary of the outcomes mentioned in the literature on good as well as poor listening. The table is organized by type of literature (i.e., communication, workplace, and healthcare research) and by the nature of the outcomes (i.e., affective, cognitive, behavioral, and relational). Articles marked with asterisks explicitly mention speaker outcomes associated with poor-quality listening, with grey highlighting used to indicate these outcomes. The literature in table 1 does not constitute a comprehensive list of articles. A systematic search could not be conducted since many articles simply refer in passing to outcomes of poor listening.

**Table 1.** Articles in fields of literature mentioning speaker outcomes arising from high- and poor-quality listening.*

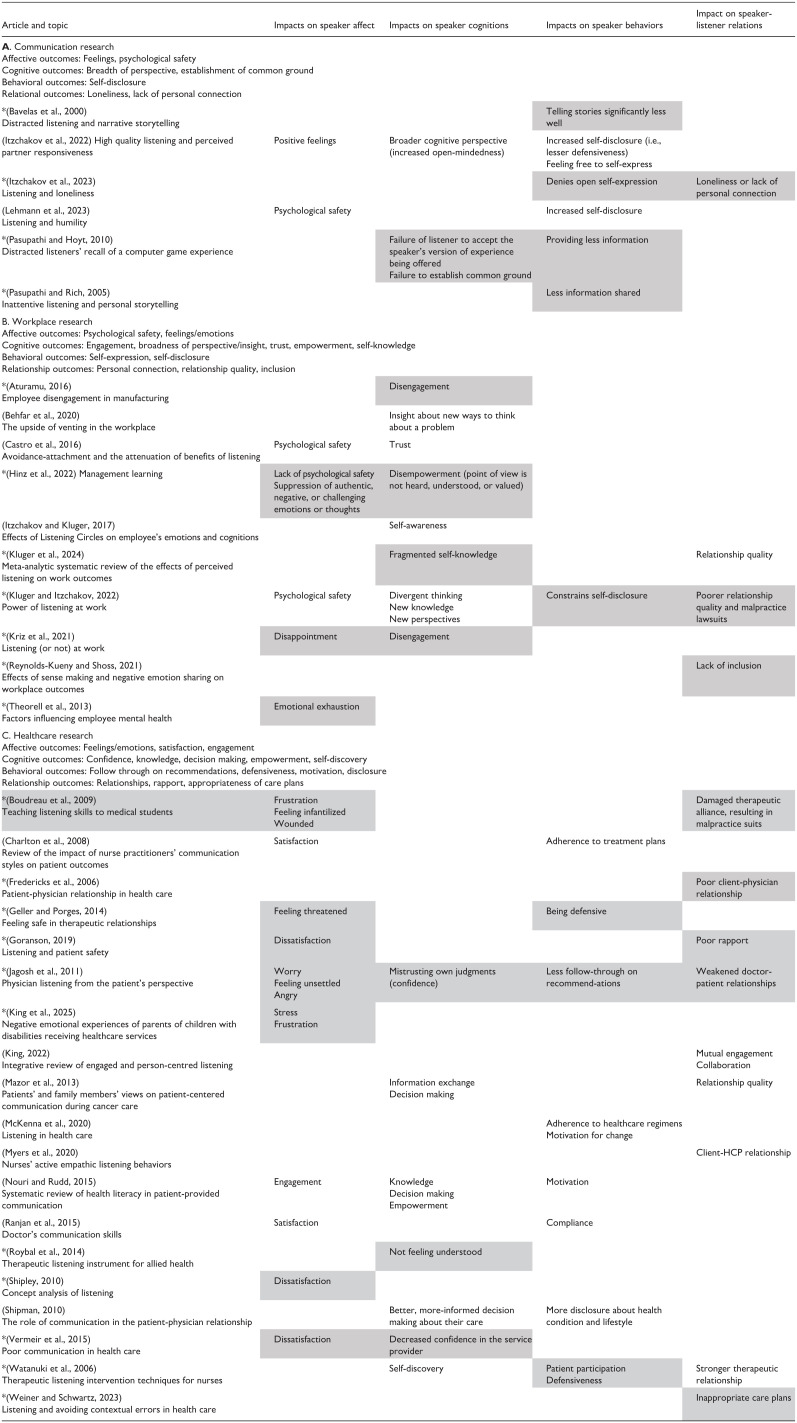

* Articles marked with asterisks contain explicit mention of speaker outcomes associated with poor listening; these negative outcomes are highlighted in grey in the table.

*Note*. Terms used by authors appear in the table.

#### ABC outcomes

For each field of literature, table 1 presents a summary of demonstrated ABC and relational outcomes, stated in neutral terms. For example, the affective outcome ‘feelings’ encompasses both positive and negative feelings. These outcomes are extrapolations of the findings in each field of literature.

With respect to **affective outcomes**, each field of research has referred to feelings and emotions, although the negative emotions mentioned in the healthcare literature are more varied. These include frustration, anger, worry, and stress, and feeling infantilized, threated, misunderstood, dissatisfied, unsettled, and mistrustful. The healthcare service delivery context understandably deals with intense, negative emotions, as there are power differences between clients and providers. Furthermore, clients and their families have expectations about the services they will receive and how they will be treated, which are often unmet. Affective outcomes are also relevant to the workplace context, which is similar to health care with respect to power differences and expectations.

With respect to **cognitive outcomes**, the communication and workplace literatures consider changes in speakers’ breadth of perspective when they experience good listening. In contrast to the communication literature, changes in empowerment and self-knowledge are discussed in the workplace and healthcare literatures (see table 1). Healthcare research differs from workplace research in noting impacts on decision making and knowledge in general, not just self-knowledge.

With respect to **behavioral outcomes**, articles in the communication and workplace literatures consider self-disclosure to be a predominant outcome of good listening, whereas the healthcare literature notes a series of negative outcomes of poor listening, including defensiveness and a lack of follow-through on recommendations (also referred to as lack of adherence or compliance).

Last, it is noteworthy that the communication literature does not typically refer to **relational** outcomes (Itzchakov et al.’s (2023) study of social rejection and loneliness is a noteworthy exception). This may be due to the focus on the listener in this field, as opposed to the speaker-listener interaction, and also the use of unacquainted dyads (with the exception of research focused on intimacy, which is not included in table 1 as the focus is on self-disclosure rather than listening per se). The workplace literature stresses relationship quality, personal connection, and inclusion as important relational outcomes. In comparison, healthcare articles have considered poor rapport and damaged relationships arising from poor listening, along with the increased likelihood of malpractice suits. In summary, contextual features of the fields of study clearly play a role in the outcomes reported to emanate from listening. These outcomes can be captured within an ABC framework, with the addition of relational outcomes.

#### Specific study findings

After each ABC summary, table 1 presents the findings from relevant studies in each field of research. These demonstrated impacts have both similarities and differences: although some of the outcomes from studies of good and poor listening are clear opposites (e.g., *increased self-disclosure* vs *lack of open self-expression*), other outcomes are qualitatively different (i.e., a difference in the kind or type of something).

For example, in the communication literature, the study by Pasupathi and Hoyt (2010) on poor listening found evidence of *failure to establish common ground*, whereas studies on good listening did not provide evidence of the opposite (i.e., *finding common ground*). In the workplace literature, the article by Hinz et al. (2022) mentioned *disempowerment* as a cognitive outcome of poor listening, whereas the studies of good listening did not report evidence for *empowerment*. In the healthcare literature, *negative emotions* (e.g., frustration, worry, stress, and anger), *decreased confidence in the service provider*, and *not feeling understood* were reported in studies of poor listening but their opposites (i.e., *positive emotions*, *increased confidence in the service provider*, and *feeling understood*) were not reported in studies of good listening.

What do these findings mean? Taken at face value, they indicate that the outcomes of good and poor listening in specific contexts may not only be the opposite of one another, but can reflect qualitative differences, in line with a conceptualization of good and poor listening as representing the end-points on a bipolar continuum of listening quality.

Of most relevance to this article, there is evidence that a diverse set of negative outcomes can arise from perceptions of poor listening, including failure to establish common ground, less self-disclosure of information, lack of psychological safety, disengagement, disempowerment, fragmented self-knowledge, lack of personal connection (loneliness), poorer relationship quality or damaged/poor/weakened relationships, lack of inclusion, negative feelings/emotions, lack of follow-through on recommendations, poor rapport, and inappropriate care plans.

### A conceptual framework of client reactions to poor-quality listening in healthcare conversations

**Figure 1.**
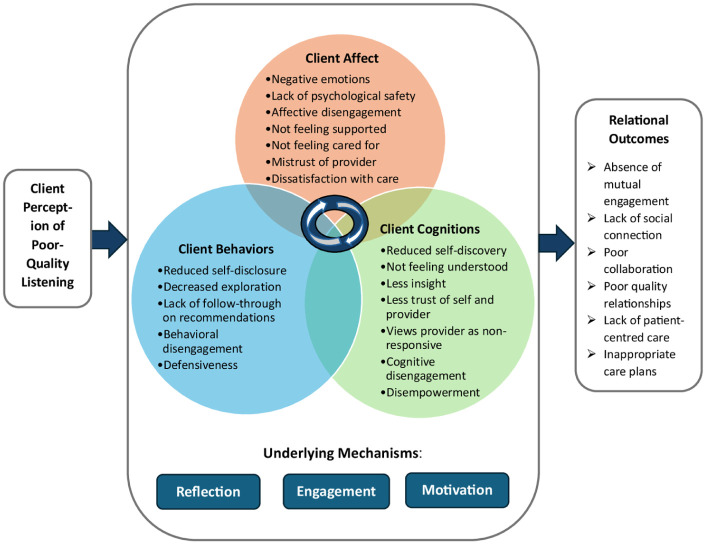
Clients’ Affective, Cognitive, and Behavioral Reactions to Perceived Poor-Quality Listening in Healthcare Conversations: A Conceptual Framework.

A holistic, multidimensional framework of the effects of poor-quality listening for healthcare clients is proposed (figure 1), based on findings of this critical review. The figure illustrates the ABC effects of poor listening in health care, based on direct evidence and extrapolation from the communication and workplace literatures. Figure 1 is also based on Itzchakov et al.’s (2022) model of listening in the workplace, which describes a process from *listening* to *perceived partner responsiveness* to *ABC effects* on the speaker. The figure illustrates how client perceptions of poor listening by a HCP can lead to multifaceted ABC reactions, by virtue of underlying mechanisms of reflection, engagement, and motivation, resulting in a variety of undesirable relational outcomes, such as poor-quality relationships with HCPs and a lack of person-centered care. The sections below describe the key elements of the framework, namely cascading ABC effects and mechanisms underlying these client reactions. ‘Cascading’ refers to a series of responses or outcomes (Kluger and Itzchakov, 2022; Fredrickson, 2001).

#### Negative effects of perceived poor-quality listening in health care

An ABC framework has not been applied previously to elucidate the nature of the effects of poor listening on the person who does not ‘feel heard’. Figure 1 links poor listening to client outcomes concerning affect, behavior, and cognitions. Thus, inferences arising from perceptions of poor listening may lead to emotions, other cognitions, and behavioral outcomes. As shown in figure 1 and table 1, some reactions are behavioral (e.g., reduced self-disclosure, decreased exploration, lack of follow-through on recommendations), some are cognitive (e.g., not feeling understood), and some are affective (e.g., negative emotional experiences, affective disengagement, mistrust of HCP, dissatisfaction with care).

Based on perceived poor-quality listening, clients can infer that the HCP is not engaged or not responsive to their concerns. As well, the subjective experience of not being listened to can lead to various inferences about listeners themselves, such as being inauthentic, neglectful, disrespectful, indifferent, not caring, pre-occupied, and/or disengaged, and perhaps even arrogant, critical, antagonistic, disagreeable, or judgmental (Itzchakov et al., 2022; Pasupathi and Hoyt, 2010). Thus, perceived poor-quality listening may trigger negative emotions, disengagement, and/or avoidance of further healthcare interactions. Clients may experience negative emotions (e.g., stress, frustration), feel judged or disrespected, form negative attitudes towards the HCP, and thus disengage affectively, cognitively, and behaviorally. It is easy for clients to detect a lack of authenticity, engagement, and/or caring on the part of HCPs (Jagosh et al., 2011).

#### Cascading negative emotions, cognitions, and behaviors

Itzchakov et al. (2022) view high-quality listening as leading to a series of cascading outcomes. Similarly, we propose that client perceptions of poor-quality listening involve cascading effects across emotions, cognitions, and behavior (Schum, 1980). Thus, one inference can lead to another, and an affective response such as feeling frustrated can lead to behavioral effects, such as avoidance, and also to inferences concerning the intentionality of the HCP. An important feature of inferences is that the logical connection between what is observed (i.e., the HCP’s listening behavior) and a person’s conclusions or deductions is frequently indirect, involving various progressions of linked ideas and reflections.

Importantly, the demonstrated outcomes of poor listening are not always the opposite of outcomes of good listening, but are sometimes qualitatively different. Furthermore, the complex nature of the ABC effects means that any attempt to specify a particular causal sequence to describe the effects of poor listening would be inadequate. The influence of cognitions and emotions on behavior and behavioral intentions are well known (Lench et al., 2013). Cognition and affect also have reciprocal effects, as thoughts shape feelings and feelings shape thoughts (Isen, 1991). Thus, cognitions and emotions are intertwined, with cognitive appraisals having the power to trigger emotions (Fredrickson, 2001), which then influence inferences and inform judgments (Clore and Huntsinger, 2007). For example, negative emotions have been found to shrink the array of thoughts and actions that come to mind (Fredrickson, 2001).

#### Hypothesized mechanisms underlying client reactions to perceived poor-quality listening

This section considers the mechanisms by which perceived poor-quality listening is hypothesized to lead to negative effects for healthcare clients. These include the mechanisms of *reflection*, *engagement*, and *motivation*. First, *reflection*, defined as the process of internally examining and exploring an issue, is a primary cognitive mechanism of learning, related to client self-discovery (Schwellnus et al., 2015; Hinz et al., 2022). As a process, reflection refers to actively and intentionally thinking critically about an experience—that is, examining one’s thoughts, feelings, and actions. Thus, client reflection on the listening conversation is considered to underlie their reactions to perceived poor-quality listening.

Second, *engagement* is a fundamental mechanism of therapeutic change (King et al., 2021), where the HCP creates a positive therapeutic context with the power to mobilize client change (King, 2017). Engagement is defined as an optimal client state comprising a hopeful stance (affective involvement), conviction with respect to the appropriateness of intervention goals and processes (cognitive involvement), and confidence in personal ability to carry out the intervention plan (behavioral involvement) (King et al., 2014). In an engaged state, clients are highly motivated to work toward therapy goals. Thus, client engagement is considered to underlie their reactions to poor-quality listening, as clients’ lack of engagement can lead to negative thoughts, feelings, and actions.

Affective engagement refers to the client’s emotional involvement in the therapy process and their connection with the HCP (King et al., 2014). Thus, mutual or reciprocal affective engagement is similar to partner responsiveness in the workplace literature (Itzchakov et al., 2022), which has been found to play a central role in mediating the effects of high-quality listening on the speaker. HCPs can harness engagement by listening and communicating effectively, adopting the perspective of the client and/or family, and being aware of, anticipating, and responding to signs of engagement and disengagement (King et al., 2021).

Third, *motivation* is a mechanism of change implicated in client engagement (King et al., 2014) and models of behavior change (Michie et al., 2014). Decreased client motivation, resulting from perceived poor-quality listening, is linked with disclosing less, shutting down, not following through, and other signs of behavioral disengagement.

## Discussion

This critical review aimed to identify what is known about the effects of perceived poor-quality listening and to synthesize this knowledge to inform research and practice in health care. Due to the scant healthcare literature, findings from communication research and listening in the workplace were also considered. This discussion considers the fields of literature that have examined listening, observations about listening-related theories and methodologies of the existing research, the nature of the evidence for negative effects of poor listening, and the mechanisms proposed to underlie the effects of poor listening.

Three fields of literature have investigated the effects of listening. Communication research has typically adopted the perspective of the listener, whereas the workplace and healthcare literatures have considered the effects on the speaker. This may reflect the fact that the workplace and healthcare literatures more often adopt a relational perspective, where good listening is considered optimal and involves the interaction between two people in a specific context.

Overall, research on listening lacks a strong theoretical basis. The review noted four mini- or small-scale theories linking listening to phenomena such as partner responsiveness (Itzchakov et al., 2022) and client engagement (Wolvin, 2010). Wolvin’s (2010) Engagement Theory of Listening intersects theoretical perspectives on listening and engagement, proposing the idea of the ‘engaged listener’ and a process view of listening behavior as a multidimensional communication phenomenon. Itzchakov et al. (2022) proposed an integrative model of high-quality listening relevant to interpersonal communication, which outlines beneficial ABC outcomes mediated by perceived partner responsiveness. Kluger and Itzchakov’s (2022) Episodic Listening Theory highlights the central importance of an episode of togetherness between two people in the workplace, generated by good quality and authentic listening, which leads to divergent thinking and the co-creation of new knowledge. Last, King et al. (2022) proposed a conceptual framework of Effective Listening in Healthcare Conversations, based on a relational perspective. This framework views listening in healthcare as a relational process that is fostered by the ‘engaged and person-centered listener’, resulting in relational outcomes such as mutual engagement and collaboration.

Each of these theories is relational and each focuses on positive outcomes of good listening. None of the theories directly target the nature of the detrimental effects of poor listening, which have been synthesized in this critical review into a framework of negative effects of poor listening in health care. In addition to direct evidence for detrimental effects of poor listening, there is indirect evidence based on logical inference from the outcomes of good listening, which have been much more extensively investigated. There appears to be an underlying assumption in the literature that poor listening is the opposite of good listening, which may partly explain why poor listening is seldom the focus of investigation. However, the direct evidence suggests that poor listening can lead not only to the absence of positive effects, but can actually be harmful, resulting in undesirable and deleterious effects on speakers. This is in line with a bipolar conceptualization of listening, with good and poor listening as positive and negative endpoints on a listening continuum, which are associated with both qualitatively and quantitatively different outcomes.

The multidimensional framework of poor listening proposed in this article is a novel contribution, developed from direct evidence and logical inference. This framework adopts the client’s perspective on the effects of poor listening. As a critical review aims to do, the framework integrates the ABC effects of poor listening in the communication, workplace, and healthcare literatures. The framework points to the role played by inferences and the mechanisms of reflection, engagement, and motivation. These mechanisms are considered to underlie the effects of perceived poor-quality listening on clients’ thoughts, feelings, and actions, and they reinforce the importance of the intentionality of the listener, especially in healthcare conversations (Jagosh et al., 2011).

### Study strengths and limitations

The strengths of this critical review include its breadth of coverage of the existing literature related to poor listening. The literature search was thorough and involved following various threads of ideas concerning the nature of cascading effects, the role of inferences, and mechanisms of change. Nonetheless, the review did not utilize a systematic or comprehensive search process. Given the lack of a substantial body of good quality evidence, the conceptual framework partly relied on logical inferences derived from demonstrated benefits of good listening.

### Implications for healthcare research

Although investigation of the framework of ABC reactions to poor listening is required, future research can be guided by this framework. This critical review and conceptual integration raise new questions and directions for research about poor listening in health care. More work is needed to examine the importance of the mechanisms hypothesized to underlie the effects of poor listening. Experimental studies are not optimal for uncovering mechanisms of change, which are better elucidated in qualitative studies targeting the perceptions, reactions, and responses of speakers who are not listened to in a particular context. As well, the review of the literature suggested the importance of conducting qualitative studies to explore factors and mechanisms related to the effects of perceived poor-quality listening on healthcare clients. At present, we do not truly understand the psychosocial dynamics that are operating, including how the client views the intentionality and authenticity of the HCP, as well as factors affecting their accuracy in inferring the intentionality of the HCP. Harrington, Noble, and Newman (2004) indicated the importance of future research to identify which clients derive the greatest benefit from a communication intervention, and to examine the impact of interventions on longer-term clinical outcomes.

### Implications for healthcare practice

For HCPs themselves, the implications of not being fully present or attentive to the client are far-reaching, as they include medical errors (Goranson, 2019), wasted time, inappropriately targeted treatments and care plans (Weiner and Schwartz, 2023), mistrust, and poor relationships with clients (Fredericks et al., 2006). By listening well, HCPs can create conditions that encourage client reflection, engagement, and motivation. Since listening is an important mechanism for facilitating high-quality connections and positive relationships, it is important to provide HCPs with training in how to be person-centered and focused on the joint or mutual interaction, not just on their own listening behaviors (Hinz et al., 2022; King et al., 2017). It is essential for HCPs to be aware of issues of power in health care. Particularly for vulnerable families of children with disabilities, who may be new immigrants or have a history of trauma, it is important for HCPs to be aware of, and fully appreciate, the potential consequences of poor listening.

## Conclusions

Listening is not just a ‘would be nice’ aspect of healthcare services, but an essential aspect of care delivery, necessary for the development of meaningful therapeutic relationships (Roybal et al., 2014). When clients feel they are not being listened to, this can have serious, wide-ranging, and cascading effects on their behavior, thoughts, and emotions, as well as on their relationships with HCPs, leading to poor collaboration, poor-quality relationships, and a lack of person-centered care.
